# Supplementary material for: Public perceptions of biospecimen sampling and uncertainty in the context of personalised nutrition
Source: PLoS One. 2025 Nov 4;20(11):e0335733. doi: 10.1371/journal.pone.0335733 (PMC12585075; doi:10.1371/journal.pone.0335733)
Supplement: S1 Table — (PDF) [file pone.0335733.s001.pdf]

(*N* = 261)	N (%)
**Age**	
16-24	18 (6.9)
25-34	42 (16.1)
35-44	69 (26.4)
45-54	40 (15.3)
55-64	66 (25.3)
65-74	21 (10.6)
75+	10 (8)
Missing	5 (1.9)
**Gender**	
Male	125 (47.9)
Female	135 (51.7)
Non-binary/third gender	31 (0.4)
**Highest academic qualification**	
No qualification	2 (0.8)
GCSE or equivalent	30 (11.5)
A level or equivalent	64 (24.5)
Undergraduate degree	106 (40.6)
Postgraduate degree	46 (17.6)
PhD/Doctorate	12 (4.6)
Missing	1 (0.4)
**Employment status**	
Employed	145 (55.6)
Self-employed	35 (13.4)
Student	19 (7.3)
No currently employed	60 (23)
Missing	2 (0.8)
**Personal annual income**	
Less than £20,000	108 (41.4)
£20,000-£30,000	58 (22.2)
£30,000-£40,000	40 (15.3)
£40,000-£50,000	29 (11.1)
£50,000-£60,000	14 (5.4)
£60,000 or more	12 (4.6)
**General health status**	
Very good	39 (14.9)
Good	128 (49)
Fair	79 (30.3)
Bad	14 (5.4)
Very bad	1 (0.4)
**Satisfaction with life as a whole**	
0 (Extremely dissatisfied)	1 (0.4)
1	2 (0.8)
2	11 (4.2)
3	15 (5.7)

4	21 (8)
5	36 (13.8)
6	41 (15.7)
7	57 (21.8)
8	50 (19.2)
9	22 (8.4)
10 (extremely satisfied)	4 (1.5)
Missing	1 (0.4)
**Self-rated overall diet in terms of healthiness**	
Very good	21 (8)
Good	104 (39.8)
Fair	106 (40.6)
Bad	27 (10.3)
Very bad	3 (1.1)
