# Supplementary material for: Public perceptions of biospecimen sampling and uncertainty in the context of personalised nutrition
Source: PLoS One. 2025 Nov 4;20(11):e0335733. doi: 10.1371/journal.pone.0335733 (PMC12585075; doi:10.1371/journal.pone.0335733)
Supplement: Attachment — Submitted filename: PONE-D-25-20637 Peer Review Critique.docx [file pone.0335733.s002.docx]

**Title:** *Public perceptions of biospecimen sampling and uncertainty in the context of personalised nutrition* (PONE-D-25-20637)

**Summary:** This paper sought to conduct a quasi-experimental study to understand the impact of certainty and sample type on responses and attitudes to personalized nutrition.

***Reviewer’s Comments:***

**Overall:**

1. This is an excellent paper investigating an under-studied topic part of precision nutrition research, that is important to understand in order to achieve success with PN based interventions.

**Introduction**

1. Page 3, Line 80-87: The authors discuss multiple definitions of PN, but do not formally define PN for their study. Perhaps this is by design but it would be good to explicitly state that the authors are not going to define PN for use in this study, and rather the study aims to assess the pre-existing conceptualization of “personalized nutrition” at this point, without defining it. (See comment 4).
2. Page 5, Line 140-155: It is true that PN is a relatively nascent field (as noted in the Discussion) and there is a lot of uncertainty, but several human studies exist that the authors could cite as adding to the complexity. For example, Zeevi et al. 2015 (Cell) is motivating the NIH-funded Nutrition for Precision Health study in the United States, which is currently ongoing and aims to enroll 8000+ people to test responses to diet. Popp et al. 2022 (JAMA) tried to apply the same algorithm used in Zeevi et al. 2015 (Cell) but for weight loss rather than glucose control, and found null results unlike Zeevi which is an interesting discussion point. The PREDICT-1 and other PREDICT studies were also done in thousands of humans. Discussion of these and other studies, even if to highlight the uncertainties these studies may raise in terms of human work, should be considered for this paragraph.

**Methodology & Results**

3. Page 8, Lines 238: How were participants recruited – targeted emails, a list serv, posters?
4. Page 8, Lines 238: How do you know participants are nationally representative?
5. Page 8, Line 248: What is a median age of 45-54? The median should be one number if representing the 261 individuals. Is this the IQR? Or is this 2 medians for the 2 different studies?
6. Either in main text or supplementary material, it would be helpful to have a table of characteristics for the participants of this study (unless age was the only demographic characteristic collected).
7. Page 8, Lines 238-248: Did you assess the level of participants’ baseline experience or knowledge with PN prior to the study? Such as, have they used mobile apps purporting to be based on precision nutrition? Was personalized nutrition defined for them in some way? I’m curious if participants understood what was meant by personalized nutrition – as simply one’s nutritional needs for height, weight, sex, physical activity – or with the additional variables mentioned in the introduction section that get closer to true individualization? Additionally, education level may influence results – see comment 8 below. I might hypothesize that those who say PN is ‘certain’ may not be as well educated about PN or nutrition in general?
8. Page 8, Lines 247-248: Participant median age of 45-54 – it would be interesting to break down the results by age group and see if there are differences between younger and older groups, considering openness to new technologies or concepts varying with age (I say this anecdotally)
9. Page 12: A Kruskal Wallis test is meant to test for differences between medians, not means. Elsewhere “median” is noted, though, so please check this is consistent throughout.
10. It would be useful to perhaps in Supplementary material, include the NHS guidelines for collecting the samples especially since the authors adapted them for this study. Adapted in what way? Identifying or accessing the specific guidelines used might not be straightforward for those outside the UK.

**Discussion:**

11. Regarding comment 7, I see a lack of background info is noted in the Strengths and Limitations – it might be good to also note the types of characteristics that would be relevant to measure, and how they might be impact the results, even though you did not measure this.

**References:**

12. Again please cite the major human studies in the field including Zeevi et al, etc. (see above).
