# Supplementary material for: Public perceptions of biospecimen sampling and uncertainty in the context of personalised nutrition
Source: PLoS One. 2025 Nov 4;20(11):e0335733. doi: 10.1371/journal.pone.0335733 (PMC12585075; doi:10.1371/journal.pone.0335733)
Supplement: Attachment — Submitted filename: Response to reviewers.docx [file pone.0335733.s004.docx]

Department of Psychology

University of Bath

Claverton Down

Bath

BA2 7AY

5 October 2025

Dear Editors,

Thank you for giving us the opportunity to resubmit our manuscript and to the reviewer for their helpful comments. Please see in the table below our response to each of the comments. If you have any questions, please do get in touch.

Best wishes,

Katharine Lee

| **Comment** | **Response** |
| --- | --- |
| ***General comments*** |  |
| Please provide additional details regarding participant consent. In the ethics statement in the Methods and online submission information, please ensure that you have specified what type you obtained (for instance, written or verbal, and if verbal, how it was documented and witnessed). If your study included minors, state whether you obtained consent from parents or guardians. If the need for consent was waived by the ethics committee, please include this information | We obtained consent via the Prolific platform. If participants did not tick the consent boxes, they did not progress to participation in the study. We have stated this in the ethics section in the methods and online submission information. |
| Thank you for stating the following financial disclosure:  “This project has received funding from the European Union’s Horizon 2020 research and innovation programme under grant agreement No 816303”  Please state what role the funders took in the study.  If the funders had no role, please state: "The funders had no role in study design, data collection and analysis, decision to publish, or preparation of the manuscript." | We have added the statement. |
| Thank you for stating the following in your Competing Interests section:  “NO authors have competing interests”  Please complete your Competing Interests on the online submission form to state any Competing Interests. If you have no competing interests, please state "The authors have declared that no competing interests exist.", as detailed online in our guide for authors at <http://journals.plos.org/plosone/s/submit-now>   This information should be included in your cover letter; we will change the online submission form on your behalf. | We have added the statement in the cover letter. |
| When completing the data availability statement of the submission form, you indicated that you will make your data available on acceptance. We strongly recommend all authors decide on a data sharing plan before acceptance, as the process can be lengthy and hold up publication timelines. Please note that, though access restrictions are acceptable now, your entire data will need to be made freely accessible if your manuscript is accepted for publication. This policy applies to all data except where public deposition would breach compliance with the protocol approved by your research ethics board. If you are unable to adhere to our open data policy, please kindly revise your statement to explain your reasoning and we will seek the editor's input on an exemption. Please be assured that, once you have provided your new statement, the assessment of your exemption will not hold up the peer review process. | Participants consented to their data potentially being used for future research.  If the paper is accepted, we will share data via the University of Bath Research Data Archive. The data access statement will be: https://doi.org/10.15125/BATH-01605.  This will be activated when we make the data available. |
| PLOS requires an ORCID iD for the corresponding author in Editorial Manager on papers submitted after December 6th, 2016. Please ensure that you have an ORCID iD and that it is validated in Editorial Manager. To do this, go to ‘Update my Information’ (in the upper left-hand corner of the main menu), and click on the Fetch/Validate link next to the ORCID field. This will take you to the ORCID site and allow you to create a new iD or authenticate a pre-existing iD in Editorial Manager. | I have added my ORCID iD as requested. |
| If the reviewer comments include a recommendation to cite specific previously published works, please review and evaluate these publications to determine whether they are relevant and should be cited. There is no requirement to cite these works unless the editor has indicated otherwise.   Please review your reference list to ensure that it is complete and correct. If you have cited papers that have been retracted, please include the rationale for doing so in the manuscript text, or remove these references and replace them with relevant current references. Any changes to the reference list should be mentioned in the rebuttal letter that accompanies your revised manuscript. If you need to cite a retracted article, indicate the article’s retracted status in the References list and also include a citation and full reference for the retraction notice. | We have evaluated the recommendations and include these citations where we feel they are most relevant, as outlined in our response to Reviewer comment 3, below.  To the best of our knowledge, we have not referenced any retracted article. |
| ***Reviewer comments*** |  |
| This is an excellent paper investigating an under-studied topic part of precision nutrition research, that is important to understand in order to achieve success with PN based interventions. Introduction | Thank you very much for this comment and for your further helpful comments. |
| Page 3, Line 80-87: The authors discuss multiple definitions of PN, but do not formally define PN for their study. Perhaps this is by design but it would be good to explicitly state that the authors are not going to define PN for use in this study, and rather the study aims to assess the pre-existing conceptualization of “personalized nutrition” at this point, without defining it. (See comment 4). | We have outlined that Ordovas suggests a tripartite approach (page 3, line 82) and then in the method explained that our explanation of metabotyping incorporated these three elements of 1) identifying individual characteristics leading to 2) targeted nutritional advice with the aim of 3) improving health. |
| Page 5, Line 140-155: It is true that PN is a relatively nascent field (as noted in the Discussion) and there is a lot of uncertainty, but several human studies exist that the authors could cite as adding to the complexity. For example, Zeevi et al. 2015 (Cell) is motivating the NIH-funded Nutrition for Precision Health study in the United States, which is currently ongoing and aims to enroll 8000+ people to test responses to diet. Popp et al. 2022 (JAMA) tried to apply the same algorithm used in Zeevi et al. 2015 (Cell) but for weight loss rather than glucose control, and found null results unlike Zeevi which is an interesting discussion point. The PREDICT-1 and other PREDICT studies were also done in thousands of humans. Discussion of these and other studies, even if to highlight the uncertainties these studies may raise in terms of human work, should be considered for this paragraph. | Thank you for highlighting these papers. We have included them in the location we feel is most relevant (lines 28-32), where the two papers are cited to underscore the point that there is a lack of clarity about whether personalised nutritional advice is more helpful than general. |
| . Page 8, Lines 238: How were participants recruited – targeted emails, a list serv, posters? | Participants were recruited via Prolific. We have explained in the text that Prolific is a platform that helps researchers recruit participants for their online research. |
| Page 8, Lines 238: How do you know participants are nationally representative? | It is possible to buy nationally representative samples from Prolific, which we did, we have added this into the text in the ‘Sample’ section. |
| Page 8, Line 248: What is a median age of 45-54? The median should be one number if representing the 261 individuals. Is this the IQR? Or is this 2 medians for the 2 different studies? | We have now included a demographic table that outlines the spread of age and have removed the reference to median age. |
| Either in main text or supplementary material, it would be helpful to have a table of characteristics for the participants of this study (unless age was the only demographic characteristic collected). | We have uploaded a supplementary file containing a table of participant characteristics. |
| Page 8, Lines 238-248: Did you assess the level of participants’ baseline experience or knowledge with PN prior to the study? Such as, have they used mobile apps purporting to be based on precision nutrition? Was personalized nutrition defined for them in some way? I’m curious if participants understood what was meant by personalized nutrition – as simply one’s nutritional needs for height, weight, sex, physical activity – or with the additional variables mentioned in the introduction section that get closer to true individualization? Additionally, education level may influence results – see comment 8 below. I might hypothesize that those who say PN is ‘certain’ may not be as well educated about PN or nutrition in general? | We did assess prior knowledge of PN as we did not want to pre-empt participants. Nor did we focus on demographic differences as we did not hypothesize about these. However, in the limitations / future research section, we have suggested that this could be a relevant consideration for future research. |
| Page 8, Lines 247-248: Participant median age of 45-54 – it would be interesting to break down the results by age group and see if there are differences between younger and older groups, considering openness to new technologies or concepts varying with age (I say this anecdotally) | We did not set out to focus on demographic differences. As per the comment above, we have highlighted that this may be something to focus on in future research. |
| Page 12: A Kruskal Wallis test is meant to test for differences between medians, not means. Elsewhere “median” is noted, though, so please check this is consistent throughout. | Thank you for this point. We have corrected to median where reporting KW test results. |
| It would be useful to perhaps in Supplementary material, include the NHS guidelines for collecting the samples especially since the authors adapted them for this study. Adapted in what way? Identifying or accessing the specific guidelines used might not be straightforward for those outside the UK. | We have noted that we adapted (shortened) NHS Trust (National Health Service UK) guidelines for collecting the three samples. We presented these to participants in the simplest way possible, to avoid participants having to read through very lengthy descriptions. |
| Regarding comment 7, I see a lack of background info is noted in the Strengths and Limitations – it might be good to also note the types of characteristics that would be relevant to measure, and how they might be impact the results, even though you did not measure this. | This has now been addressed as per comment 9. |
| Again please cite the major human studies in the field including Zeevi et al, etc. (see above). | We have included Zeevi and Popp references, as noted above in point 3 |
